# On-demand electrochemically controlled compound release from an ultrasonically powered implant†

**DOI:** 10.1039/d2ra03422k

**Published:** 2022-08-17

**Authors:** Max L. Wang, Christian F. Chamberlayne, Haixia Xu, Mohammad Mofidfar, Spyridon Baltsavias, Justin P. Annes, Richard N. Zare, Amin Arbabian

**Affiliations:** Department of Electrical Engineering, Stanford University Stanford CA USA arbabian@stanford.edu; Department of Chemistry, Stanford University Stanford CA USA; Department of Medicine, Division of Endocrinology, Stanford University Stanford CA USA

## Abstract

On-demand drug delivery systems are promising for a wide range of therapeutic applications. When combined with wireless implants for controlled drug delivery, they can reduce overall dosage and side effects. Here, we demonstrate release of fluorescein from a novel on-demand release system for negatively charged compounds. The release system is based on a modified electroresponsive polypyrrole nanoparticulate film designed to minimize ion exchange with the stored compound – a major passive leakage mechanism. We further designed an ultrasonically powered mm-sized implant to electronically control the on-demand drug delivery system *in vivo*. Release kinetics are characterized both *in vitro* and *in vivo* in mice using fluorescein as a model drug, demonstrating the feasibility of wireless, controllable drug release using an ultrasonically powered implant.

## Abstract 2

Controlled release using electroresponsive nanoparticles and an ultrasonically powered battery-free implant demonstrate a potential implantable drug delivery system platform.

## Graphical abstract

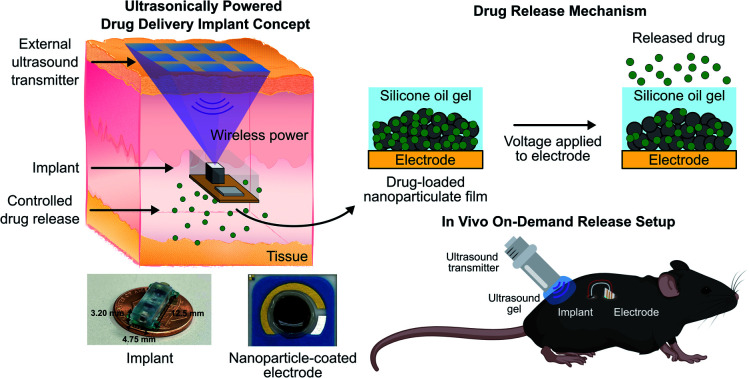

## Introduction

Electroresponsive polymers are a promising material for spatially and temporally controlled drug delivery systems (DDSs). These polymers allow for release of a target drug from an electrode coating in response to a voltage stimulus.^1,2^ When coupled with implant electronics for reliable and precise stimulus generation, they allow for controlled drug dosages to be delivered in a localized fashion, thereby reducing overall dosage, side effects, and cost. Temporal control also allows for closed-loop drug release, wherein drugs are delivered when a sensor detects a given condition. This allows for the treatment of life threatening emergencies which require immediate medication, like severe hypoglycemia caused by an insulin overdose.^3^

A range of constraints restrict the available materials, stimuli, and chemistry used in drug release mechanisms. The device needs to be biologically compatible when implanted, not degrade over time, and have chemistry that is robust against interference from the wide variety of biological molecules present in the body. In addition, the release mechanism should be applicable to a wide scope of potential drugs. Using electronics on the device to trigger drug delivery enables reliable and precise stimulus control, allowing for more fine-tuned, localized release regulation. Electrical control also allows for easy modularization of the system, wherein the sensing, system control, and drug release are all separate components that can be easily interfaced with existing technology. Many different electronically controlled release mechanisms have been investigated, including electrothermal membrane ablation,^4^ electrolytic pumping,^5^ electrochemical membrane dissolution,^6^ electrostatic channel modulation,^7^ and electrical stimulation of intrinsically conducting polymers like polypyrrole (PPy).^8^

**Fig. 1:**
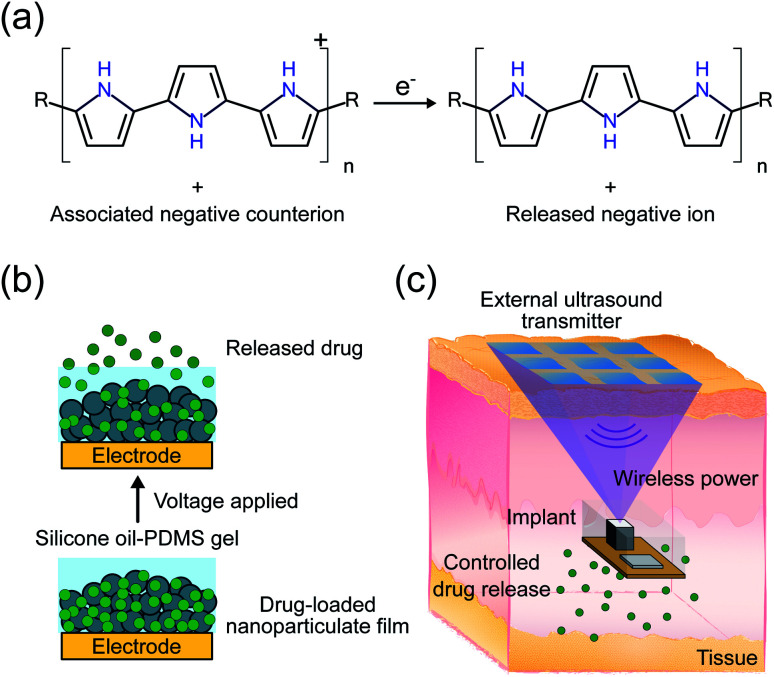
(a) Polypyrrole reduction and associated counterion release half-cell reaction. (b) Diagram of the drug release mechanism consisting of a voltage-controlled drug-loaded nanoparticulate film and silicone oil-PDMS gel. (c) Conceptual diagram of a wireless drug delivery system using an external ultrasound transmitter to power an implant with electronics to control a drug delivery module (underneath).

We propose an electroresponsive release system using a PPy nanoparticulate film in combination with an ultrasonically powered implant for wirelessly controlled drug delivery. Polypyrrole is a p-type conductive polymer that incorporates negatively charged counterions to maintain charge neutrality. A negatively charged target drug can be incorporated with the PPy as its counterion meaning that the scope is usually limited to negatively charged drug ions. A similar formulation has been demonstrated for positively charged ions but only for thin films as opposed to the nanoparticulate films used in this work.^2^ Release of the drug is accomplished by electrochemically reducing the PPy backbone. Reduction under the correct conditions causes the expulsion of the drug from the surface of the PPy (Fig. 1a).^2,9,10^ The advantages of PPy include *in vivo* stability, biocompatibility, and low actuation voltage.^11–15^ In particular, low actuation voltage mitigates drug degradation caused by the release mechanism. Thin PPy films have been utilized for releasing a variety of drugs,^2^ such as dexamethasone,^8^ methotrexate,^16^ and risperidone.^17^ However, these films suffer from two major issues: (1) limited drug loading and release capacity due to low surface area^18^ and (2) potential ion exchange with other biologically available ions leading to undesired passive drug leakage.^19^

The drug release capacity is limited because only drugs stored at the surface of the PPy can be released. The general solution is to prepare a high surface area PPy coating, and a variety of different methods have been developed to this effect.^1^ We have addressed this problem in previous work by using a high surface area film composed of PPy nanoparticles (PPy NPs).^13^ The conductivity of PPy allows for the whole structure to be in electrical contact with the electrode and the sponge-like structure allows for increased drug storage capacity and faster release. We have previously demonstrated release of drugs from this system *in vitro* at room temperature.^13^

While the PPy NP film addresses the first issue of low drug loading, the non-triggered passive release of drugs from the coating was not addressed in our previous work.^13^ We hypothesize that the passive release is mostly caused by a counterion exchange mechanism with ions in the surrounding solution, which can be significant *in vivo*. In this work, we mitigate counterion exchange through the design of the electrode and addition of a silicone oil-PDMS gel coating. In contrast to previous work on such systems, release of the target drug ion now takes place in two steps; first, reduction of the PPy backbone, as in the previous mechanism, followed by electrophoresis of the drug ion through the protective silicone oil-PDMS gel coating. Ions from the biological system cannot diffuse through the gel layer and thus should not exchange with the stored drug ions. Meanwhile, the electrophoresis step still allows for the target ions to be released out into the biological system when a voltage is applied (Fig. 1b).

Conventional implantable electronic DDSs are usually relatively bulky and require invasive surgery, meaning that they cannot be easily implanted anywhere in the body.^4^ The bottleneck for miniaturization is the large battery that is required to power the implant for years or even decades. Wireless power transfer obviates the need for a large battery, allowing the implant volume to be dictated by drug loading rather than power requirements. Wirelessly powered DDSs have been proposed using inductive power transfer, however their operation depth is constrained to around 1 cm, limited by low efficiency at larger depths.^5^ We have previously proposed using ultrasound (US) to power and control implants for a variety of applications including drug delivery.^20–22^ In this system, an external transmitter would send US waves to an implant which contains a transducer to convert the received waves into electrical power as well as electronics to control drug release (Fig. 1c). Ultrasound provides an efficient mechanism to wirelessly transfer power due to its low propagation loss, high safety limit, and focusing capabilities, meaning that implants can be placed deeply near the target area.^23^ The previous proof-of-concept DDS demonstration was sensitive to the received acoustic power, did not use an implantable form factor, and was only tested *in vitro*.^20^ The received power can be challenging to precisely control *in vivo* because it depends on implant depth and alignment which can change across individuals and over the duration of the release making the release rate unpredictable. Here we add electronics to ensure consistent release, miniaturize the device footprint, and package the electronics in a biocompatible encapsulation for implantation *in vivo*.

Combining the PPy NP drug delivery module (DDM) with US powered stimuli-controlling electronics on an implantable device provides a strong platform for precise spatiotemporal dosage control, which can maximize therapeutic efficacy while minimizing side effects (Fig. 1c).^24–26^ In this work, we demonstrate release of fluorescein (FL) from the DDM. Fluorescein is used as it is highly sensitive and specific which enables us to measure low concentrations of drug leak or release *in vivo*. The implant and DDM are first tested *in vitro* to characterize the release rates and kinetics. Finally, wirelessly powered FL delivery is demonstrated *in vivo* in adult mice.

## Experimental

### Materials

Pyrrole (reagent grade, 98%), sodium dodecyl sulfate (ReagentPlus®, ≥98.5%), iron(iii) chloride (reagent grade, 97%), fluorescein sodium, Trizma® hydrochloride buffer solution, Pur-A-Lyzer™ Dialysis Kits, Whatman® Anotop® 25 syringe filters, alginic acid sodium salt, calcium chloride (ReagentPlus®, 99.99%), and BD Falcon cell strainers (40 μm, Nylon) were purchased from Sigma-Aldrich (St. Louis, MO). Ringer's solution was purchased from Flinn Scientific (Batavia, IL). Isopropanol and silicone oil (S159-500) were purchased from Fisher Scientific (Hampton, NH). Poly(dimethylsiloxane) (PDMS, RTV 615) was purchased from Momentive (Waterford, NY). Screen printed electrodes were purchased from Metrohm (Herisau, Switzerland).

Twelve-week-old male C57BL/6J mice were purchased from Jackson Laboratories (Bar Harbor, ME) with a body weight of 26–28 g. They were maintained at 22 ± 2 °C and 50 ± 5% relative humidity with a 12 h light/dark cycle. All mice had ad libitum access to rodent chow diet and water. All animal procedures conducted in this study were reviewed and approved by the Administrative Panel on Laboratory Animal Care at Stanford University. The protocols were followed in the conduct of the animal studies.

### Electrode modification

Dropsens screen printed electrodes (DRP-C220AT) were modified with a 3D-printed 300 μm tall PLA ring around the working electrode (WE). The ring was 3D-printed using a Micro Series M3D printer as a 6 mm outer diameter (OD), 5 mm inner diameter (ID), 300 μm tall ring with a 50 μm layer height. The ring was subsequently fused around the WE of the screen printed electrode by briefly heating the electrode to 215 °C on a hot plate. All experiments with the DRP-C220AT electrodes in this work are run as two-electrode reactions with silver to silver chloride as the second half cell of the redox reaction. The silver electrode is used as the counter electrode (CE). DRP-C220AT has a gold, third electrode present (for use in three-electrode setups) which was left disconnected in all experiments.

### Nanoparticulate electrode coating fabrication

Fluorescein-loaded polypyrrole nanoparticles (FL-PPy NPs) were formed using a micelle-templated synthesis.^27^ To prepare FL-PPy NPs, 6 μL of pyrrole and 24.9 μL of a 100 mg mL^−1^ aqueous solution of fluorescein disodium salt was added to 1 mL of 0.1 M SDS in 40 mM HCl. After 30 min of stirring, 80 μL of 625 mg mL^−1^ iron(iii) chloride in water was added to the solution and the reaction mixture was stirred for 24 h at room temperature overnight. Samples were then dialyzed 3 times in excess of 24 hours in 300 mL of distilled water and dried under vacuum. Dried PPy NPs were reconstituted in a 5 mg mL^−1^ solution in isopropanol, tip sonicated to suspend the NPs (three 30 s long pulses at 6 W, 30 s apart), and aerosol spray coated onto the WE with a simple tape mask containing a 4 mm hole over the WE.

The aerosol spray device consists of a 100 μm ID, 365 μm OD silica capillary that is concentric inside a 0.020 in ID, 1/16 in OD stainless steel tubing. The end of the capillary is flush with the end of the stainless-steel tubing. The stainless-steel tubing is pressurized with 50 psi nitrogen gas to form a sheath gas for the spray; meanwhile, the FL-PPy NP containing isopropanol solution was fed at a flow rate of 5 μL min^−1^ through the capillary. The masked WE was placed 13 mm below the capillary opening.

A silicone oil-PDMS gel was made using a precursor consisting of a 1 : 15 ratio potting compound to crosslinker diluted 10× in silicone oil. A 2 μL sample was deposited inside the ring surrounding the modified WE. The electrodes were then placed under vacuum for 1.5 h to remove air bubbles, and then heated at 115 °C for 1.5 h to cross-link the gel. Just prior to use, electrodes were washed by dipping into 20 mL of deionized water three times to remove any excess silicone oil on top of the gel.

The morphology and structure of the NPs were determined by an FEI Magellan 400 XHR Scanning Electron Microscope (SEM, FEI Company, OR, USA). The NPs were mounted on an SEM stub without sputter coating before imaging.

### Implant electronics

**Fig. 2:**
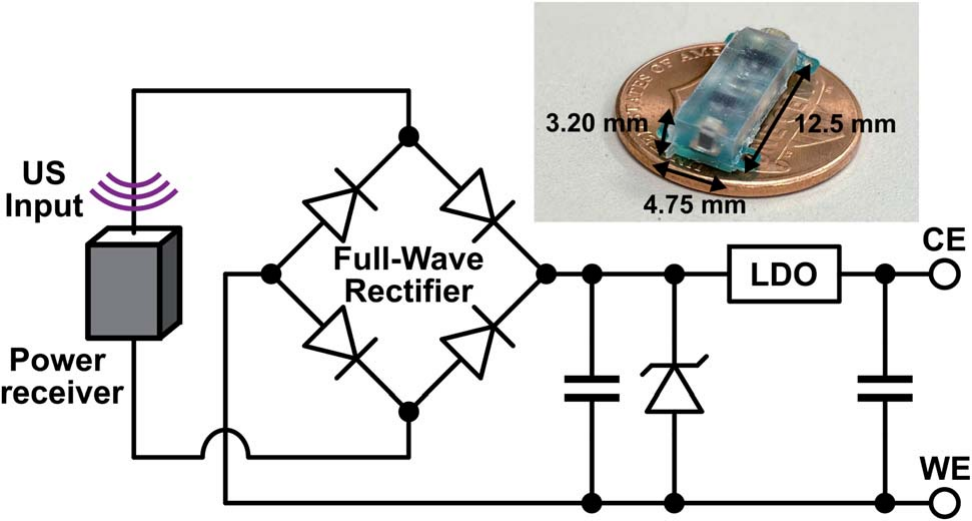
Schematic of the implant components along with a picture of the fully packaged implant without the external wires used to connect to the drug delivery module.

The implant was composed of a piezoelectric power receiver (PZT4), a full-wave rectifier for AC–DC conversion, two storage capacitors, a protection diode, and a low-dropout voltage regulator (LDO) for electrical stimulation (Fig. 2). To initiate drug release, US was transmitted by an external transducer and received by the implant piezoelectric receiver, which converted the acoustic waves into AC electrical power. The AC power was rectified by the full-wave rectifier into a DC voltage which was stored on a capacitor. In previous work,^20^ the rectified voltage was used to directly control drug release which is difficult to precisely control *in vivo*. Here, the LDO was included to regulate the rectified voltage to a constant −1 V (working electrode *vs.* counter electrode), which was sufficient for reducing the PPy NPs and stimulating drug release. The constant voltage ensured that regardless of how much acoustic power was received by the implant, the drug release would be consistent. The reverse biased zener diode protected the LDO by keeping the rectified voltage from exceeding the LDO's input voltage limits. The voltage was held until the external transducer stopped transmitting, which caused the implant to lose power and drug release to stop. The implant components were packaged on a mm-sized printed circuit board (PCB) and covered in PDMS and parylene-C to protect the electronics. Wires from the implant were used to connect to the DDM to allow for implant device reuse.

### *In vitro* leakage characterization

The FL leakage test provided information on the effects of silicone oil-PDMS gel layer on the nanoparticulate film. The DDM was placed into a test tube initially containing 2 mL of Ringer's solution or DI water at 37 °C. The test tube was capped to prevent evaporation. Test tubes were kept in a water bath at 37 °C for the duration of the experiment. We collected 150 μL Ringer's solution/DI water at each time point and then replaced it with another 150 μL of fresh Ringer's solution/DI water. First, we examined the FL leakage on electrodes with and without silicone oil-PDMS gel for 1 h to characterize the amount of leaked FL. Second, we performed a long-term leakage test to measure the leaked and released FL from electrodes with silicone oil-PDMS gel over 24 h. After 24 h, a voltage of −1 V was then applied for 10 min between the WE and the silver CE in a two-electrode setup. The −1 V potential was provided by a potentiostat (WaveNow AFTP1, Pine Research, Durham, NC). The FL concentration in each sample was determined using fluorescence detection by a microplate reader (SpectraMax iD3, Molecular Devices, LLC, San Jose, CA) with the excitation wavelength set at 480 nm and the emission at 520 nm. Fluorescein release calculations took into account the change in volume and the amount of FL removed from the test tube by previous samples.

### *In vitro* release kinetics

Following the same initial procedure as the leakage rate characterization, the electrodes were placed into a test tube initially containing 2 mL of Ringer's solution at 37 °C. They were allowed to sit in the solution for 10 min open circuit without any applied voltage. Cumulative release of FL from FL-PPy NPs was then measured at different potentiostat voltages (−0.5 V, −1.0 V, and −1.2 V) for 10 min. Fluorescein samples were collected at the same time points (0, 1, 2, 3, 5, 7, 10 min) for each controlled release. To study pulsatile release from the DDM, four cycles of electrical stimulation were applied at −1 V for 1.5 min each. Between each stimulus, the potentiostat was turned off for 1.5 min. Fluorescein samples were collected during each stimulus transition. For each time point, a 150 μL sample was taken and then 150 μL of fresh Ringer's solution was added back into the test tube. The collected samples were analyzed the same as above.

### *In vitro* release comparison

Following the same initial procedure as the leakage rate characterization, the electrodes were placed into a test tube initially containing 2 mL of Ringer's solution at 37 °C. Samples were taken by removing the electrode from the liquid, vortexing the test tube, removing a 150 μL sample to a well plate, then replacing the electrode.

Samples were taken at 0, 30, 120, and 130 min to measure the leakage. The test tube was uncapped after the 120 min time point, and the electrode was inserted into a connector cable. The electrode was allowed to sit in the solution for 10 min open circuit to act as a control. To characterize the release rate a voltage of −1 V was then applied for 10 min between the WE and the CE. A final sample was collected after the 10 min voltage stimulus.

The −1 V potential was provided by either a potentiostat or by the implant. To provide the −1 V potential, the implant was powered with an US transmitter (A303S, Olympus Corporation, Tokyo, Japan) driven by a signal generator at 1 MHz. The transmitter was separated from the implant by a 2 cm thick US gel pad (Aquaflex, Parker Laboratories Inc., Fairfield, NJ). The signal generator was turned on for the duration of the release and stopped after 10 min. The collected samples were analyzed the same as above.

### *In vivo* fluorescein absorption kinetics

A 10% fluorescein sodium solution (Ak-Fluor®, Akorn Pharmaceutical Co., Lake Forest, IL) was diluted with sterilized saline to make 100 μL working solutions at different dosages of total FL content: 10 μg, 1 μg, and 100 ng. Mice were injected subcutaneously with either the FL working solution or the same volume of saline as control. Thirty microliters of tail vein blood was collected by Na-heparinized haematocrit tubes (Cat: 51 613, Globe Scientific, Mahwah, NJ) at exact time point (0 min as before injection, 5 min, 15 min, 30 min, 60 min, 90 min, and 120 min after injection). All blood samples were centrifuged at 10 000 rpm for 15 min and 10 μL of plasma was collected for fluorescence detection.

### *In vivo* release kinetics

On surgery day, each mouse was supplemented with 0.5 mL Ringer's solution through intraperitoneal injection before being anesthetized by isoflurane inhalation. Once the surgical plane of anesthesia (respiratory rate 55–65 times per minute, absence of withdrawal reflex, mucous membrane color is pink) was reached, one or two separate incisions (about 1 cm for the electrode and 3 mm for the implant) were made at the middle line of the back skin of the mouse after hair removal. Subcutaneous pockets were then created by blunt separation in each mouse to place just the electrode or the electrode and implant depending on whether the potentiostat or the implant provided the stimulus. To ensure robust wet contact of the CE with the mouse muscle, a hydrogel was formed using a bead of 5 μL of 2% wt. sodium alginate in Ringer's solution on the silver electrode and crosslinked by submerging it in 150 μL of 0.5 M calcium chloride. The electrode was then rinsed three times in 20 mL of deionized water.

After implant and electrode insertion, a small amount of US gel (Aquasonics 100, Parker Laboratories, Inc., Fairfield, NJ) was placed over the skin above the implant and the US transmitter (connected to the signal generator) was positioned on the gel to ensure good contact to the skin. The transmitter was aligned to the implant by monitoring the implant output voltage. Ten minutes after surgery, the implant was connected to the electrode and the signal generator was turned on, triggering the US transmitter and activating the electrodes with a potential of −1 V. After 10 min of powering, the signal generator was turned off.

Tail vein blood samples were first collected immediately after surgery (recorded as 0 min), then immediately after the end of powering at 20 min followed by 25 min, 35 min, 50 min, 80 min, 110 min, and 140 min to monitor the FL kinetics in circulation. The control group went through the same procedure with electrode and implant insertion, US powering of the implant, and tail blood sample collection. The only difference was that the implant was not connected to the electrode during US powering.

For the potentiostat controlled release, only the electrode was placed in the subcutaneous pocket. Ten minutes after the surgery, the potentiostat was turned on to a potential of −1 V for 10 min. The blood samples were collected at the same time points as the implant-controlled release.

## Results and discussion

### Electrode structure and release mechanism

**Fig. 3:**
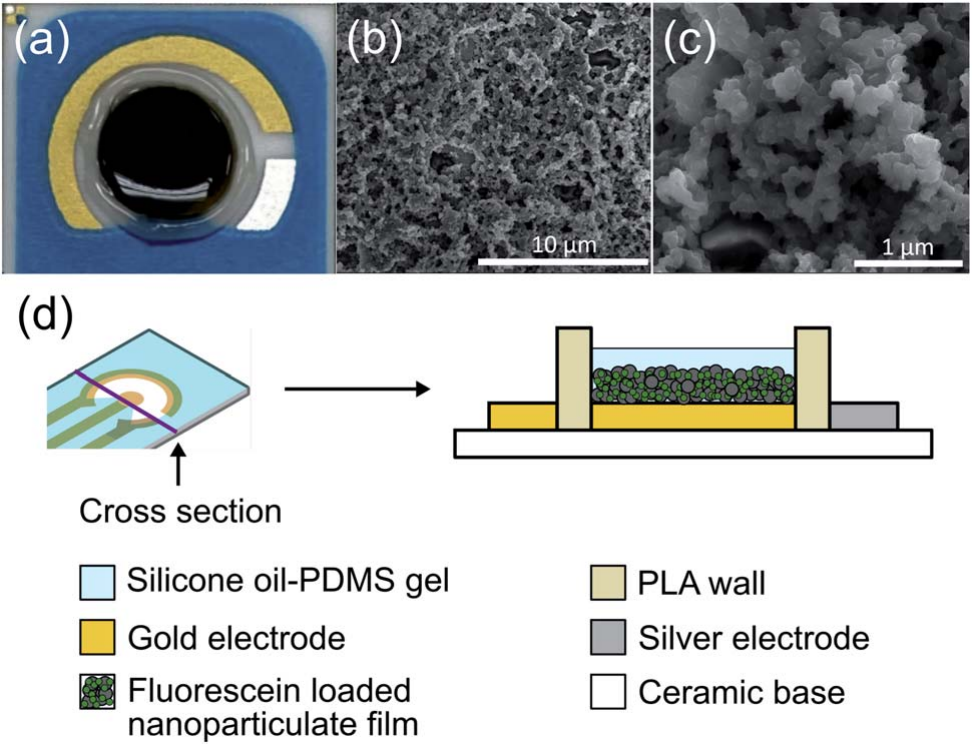
(a) Picture of the DDM electrode coated with FL-PPy NPs and silicone oil-PDMS gel (outer gold auxillary electrode is not used). (b) and (c) SEM of FL-PPy NP film on an electrode. (d) Schematic of electrode layer structure.

The drug release coating on the electrode was composed of a PPy nanoparticulate film. The film was characterized and optimized for maximizing controlled release while minimizing passive leakage. We created a higher surface area film by forming nanoparticles of FL-loaded PPy and then aggregating them into a nanoparticulate film on the electrode surface (Fig. 3). The resulting highly porous structure had a high surface area, and the conductive nature of the PPy allowed for electrical access to the whole structure. An image of the DDM can be seen in Fig. 3a. Scanning electron microscope images show the high surface area of the resulting nanoparticulate film (Fig. 3b and c). The larger pore sizes of these films have been shown to enhance drug loading and facilitate faster release.^13,27^

Passive ion exchange at the surface of the drug-loaded PPy NP film can cause undesirable release of drugs, which is a major mechanism for leakage in conductive polymer-based drug release systems. To minimize passive ion exchange at the surface, we incorporated a silicone oil-PDMS gel coating over the nanoparticulate film. We hypothesized that this insulation would prevent external ions present in the liquid surrounding the electrode from reaching the PPy surface and exchanging with the FL molecules. The electrode was surrounded by a 3D-printed wall of PLA both to protect the coating from abrasions *in vivo* and to hold the gel in place while it set during fabrication. The DDM was constructed as a two-electrode system in which the gold WE held the nanoparticulate coating and the silver CE mediated the reaction of chloride ions to silver chloride (Fig. 3d). All voltages are given as the difference between these two electrodes.

The addition of the silicone oil-PDMS gel uncovered a new challenge during the fabrication. Previous work used drop casting to deposit the nanoparticulate film.^13^ Unfortunately, this technique created an uneven PPy NP layer which resulted in inconsistent gel coverage over the nanoparticulate coating and inconsistent leakage and release rates. This was corrected using a spray coating method to form a more uniform and consistent PPy NP film. Overall we found that the designed structure allowed for more reliable electrically triggered release, while minimizing passive release from the film.

**Fig. 4:**
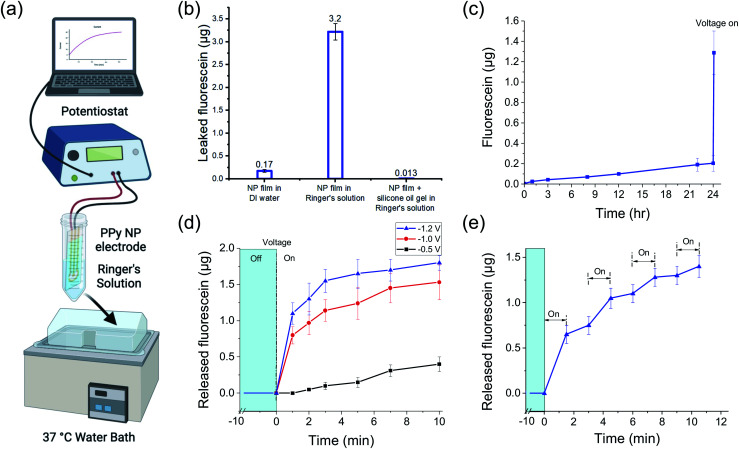
Fluorescein passive leak and active release *in vitro* from FL-loaded electrodes controlled by a potentiostat. (a) Diagram of the measurement setup. (b) Cumulative passive leak over 1 h (mean ± SD) of FL from the PPy nanoparticulate film without the silicon oil-PDMS gel coating, in DI water (left) and Ringer's solution (center), and with the silicon oil-PDMS gel coating in Ringer's solution (right). (c) Cumulative passive leak (mean ± SD) of FL from the PPy nanoparticulate film with silicone oil-PDMS gel coating over 24 h followed by electrically triggered release (−1 V for 10 min) after the 24 h time point. (d) Cumulative release (mean ± SD) of FL from the PPy nanoparticulate film during controlled release at different voltages (−0.5 V, −1.0 V, and −1.2 V) for 10 min. (e) Cumulative release (mean ± SD) of FL during pulsed release (−1 V for 1.5 min) after four cycles of on/off electrical stimulation. *N* = 3 per group for all measurements.

To characterize the passive FL leakage before and after adding the gel, the FL-PPy NP coated electrodes were soaked at 37 °C in Ringer's solution, a carbonate based buffer with the same ionic strength as interstitial fluid, without a voltage applied (Fig. 4a). When the bare FL-loaded PPy NP coating was placed into Ringer's solution, a majority of the FL was passively leaked within an hour (Fig. 4b). We attribute the majority of the leakage to an ion exchange mechanism between the bound FL and the salts in the surrounding Ringer's solution as the NPs were dialyzed to remove excess unbound FL prior to forming the coating. Further confirmation of this hypothesis was made by placement of the bare FL-loaded PPy NP coating into DI water, which showed reduced FL leakage. The observed leak of FL in Ringer's solution is greatly reduced by the addition of the silicone oil-PDMS coating which we hypothesize prevents the salt ions in the Ringer's solution from reaching, and thus exchanging with, the FL ions bound to the PPy NP coating surface.

Fig. 4c shows the continuation of the FL leak experiment from the silicone oil-PDMS coated samples in Ringer's solution out to 24 hours. At the end of the 24 h experiment, the FL release was electrically triggered using a potentiostat to demonstrate that the DDM was still functional. It is important that the addition of the silicone oil-PDMS coating, while preventing surrounding ions from reaching the PPy surface, does not prevent the triggered release of the FL ions themselves. When a negative voltage is applied to the WE, it reduces the PPy backbone and pushes the FL off the surface into the silicone oil-PDMS gel. While not normally soluble in silicone oil, the FL ion lacks a nearby counterion and thus cannot precipitate out of the solution. Gel electrophoresis then drives the FL out of the gel layer and into the surrounding liquid, provided that the gel layer is sufficiently thin and not highly crosslinked. If the gel is too highly crosslinked or too thick for the FL to traverse, charge build up at the electrode surface prevents further reduction of the polymer backbone. Different amounts of gel precursor and gel compositions were tested to find an effective coating, ultimately resulting in a gel using 1 : 15 ratio potting compound to crosslinker diluted 10× in silicone oil. Note that PPy NPs are not released from the nanoparticulate film as part of the drug release mechanism. In terms of bio-compatibility, there is a risk PPy NPs could be released into the body from a mechanical failure of the coating, and thus their biocompatibility should be considered. Fortunately, while less studied than PPy thin films, PPy NPs have also been seen to be biocompatible.^28,29^

### *In vitro* release characterization

To investigate the release kinetics of the DDM and demonstrate different release profiles, we sampled the released FL amount over the 10 min potentiostat stimulation duration. Note that the release of FL was not linear with time. Fig. 4d shows how the release amount changed over 10 min during a constant voltage stimulus at −0.5, −1.0, and −1.2 V. The total release amount increased with more negative voltage as has been shown previously.^20,27^ The increased release also corresponded to increased current, as would be expected from an electrochemical reaction (Fig. S1†). Notably, the system could tolerate the higher voltages without water splitting due to the silicone oil-PDMS layer preventing water from reaching the WE. However, applying high voltages is risky as any defects in the silicone oil-PDMS layer that allow water to come to the electrode surface will worsen as gas is generated. For the subsequent experiments, we stayed at −1 V which was within the safe potential window to avoid water splitting.

The release rate decreased over time as the stored FL was depleted with half of the total release taking place in the first minute. The silicone oil-PDMS gel complicated modeling of the release kinetics. Throughout the release, FL ions were electromigrating through the gel layer. The buildup of FL ions near the electrode surface slowed the reduction rate of the PPy polymer and also contributed to the slowing release.

Drug release across multiple pulses may be advantageous for certain applications. Pulsing the stimulation voltage generated changes in the release profile (Fig. 4e). The voltage was pulsed at −1 V for 1.5 min with 1.5 min between pulses. The release amount changed across the four release periods with each subsequent stimulus resulting in a slightly lower release amount, which was to be expected as the FL stored in the PPy NPs decreased. For more consistent pulses, the pulse duration could be decreased and/or each subsequent pulse could use more negative voltages. After each stimulus, there was residual FL in the gel that was still in the process of migrating out of the gel. This was observed as a slight FL increase during off states between pulses.

Combining the electroresponsive PPy NPs with a wireless implant allows for on-demand release *in vivo*. We designed a miniaturized US powered implant system to wirelessly control compound delivery (Fig. 2). This system included an external US transmitter for power transfer and a battery-free implant containing a piezoelectric power receiver, electronics, and a connector for the DDM. To ensure consistent release independent of received acoustic power level (which could depend on transmit power, implant depth, and alignment), the electronics rectified and regulated the received power to a constant DDM stimulation voltage. The implant dimensions were 12.5 mm × 4.75 mm × 3.20 mm for easy implantation during *in vivo* testing and can be further miniaturized depending on the application.

**Fig. 5:**
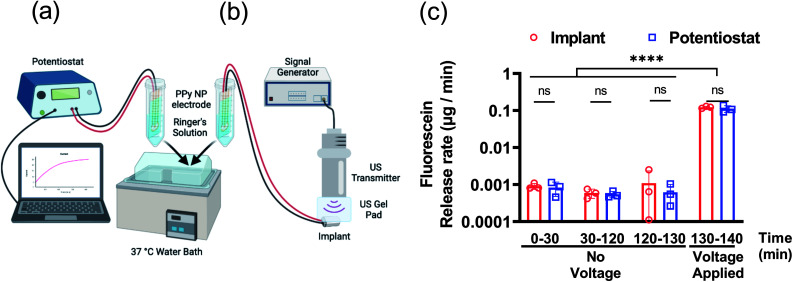
Fluorescein release *in vitro* from FL-loaded electrodes controlled by a potentiostat or implant. Diagrams of the measurement setups with (a) the potentiostat and (b) the ultrasonically powered implant. (c) Fluorescein release rate before and during voltage application. *N* = 3 per group. ns: no statistical significance. ****P < 0.0001.

Further characterization of the FL release was tested using both the potentiostat and implant to compare performance. The DDMs with FL-PPy NP coatings were placed in Ringer's solution at 37 °C to assess the release rate of FL from the coatings without a triggering voltage for 130 min, and then with a triggering voltage for 10 min. A benchtop potentiostat was used to establish a baseline for comparison to wirelessly powered release (Fig. 5a). We then demonstrated release using the US powered implant to verify that the implant could provide the necessary power to trigger release from the DDM (Fig. 5b). A time frame of roughly 2 h was chosen to match the time frame of the subsequent *in vivo* experiments.

Characterization of passive and active release rates *in vitro* with the potentiostat and implant demonstrated the consistent performance of the DDM (Fig. 5c). Measurements were taken from 0 to 30 min and 30 to 120 min to determine if the detected leak was skewed by a burst release caused by the initial wetting of the surface. The measured leak rate was approximately 1 ng min^−1^ and was consistent throughout the experiment. This is higher than the average leak rate observed in the 24 h leak experiment in Fig. 4c. It is worth noting that Fig. 4c shows an increased leak rate for the first 3 h and then continues to slow down which can explain this discrepancy.

The measurement from 120 to 130 min was used as a control consisting of the same 10 min time frame as the release. After the control, a potential of −1 V was applied to each DDM for 10 min to trigger the release of FL. The release rate during the voltage stimulation was about 100 ng min^−1^. For a 10 min stimulation, the FL release amount was approximately 1.5 μg. Given the 4 mm diameter electrode, this was a release capacity of 12 μg cm^−2^ for a 10 min release at −1 V. The results for potentiostat stimulation compared to wireless implant stimulation were nearly identical indicating that the implant could provide the necessary power to trigger release from the coating.

Characterization of the *in vitro* FL leakage and release rates as well as the kinetics across different voltages and multiple pulses, demonstrated the viability of this release mechanism for drug delivery. Furthermore, the US controlled implant was shown to be capable of providing sufficient power to stimulate the FL release.

### *In vivo* release characterization

**Fig. 6:**
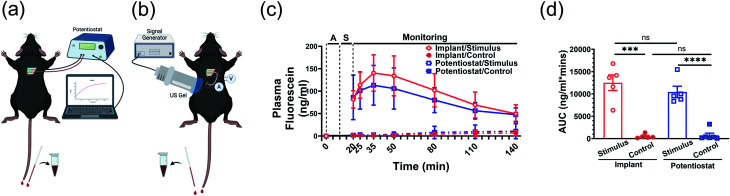
Fluorescein release in male C57BL/6J mice from FL-loaded electrodes with a potentiostat or implant. Diagrams of the measurement setup with the (a) potentiostat and (b) the ultrasonically powered implant. V: voltmeter, A: ammeter (c) Plasma FL kinetics with −1 V potential applied (stimulus) or without voltage (control). Plasma FL level at 0 min of each mouse was subtracted for background normalization. A: ultrasound alignment to implant, S: stimulus. (d) Area under curve. Data represented as mean ± SD. *N* = 5 per group. ns: no statistical significance. ***P < 0.001, ****P < 0.0001.

To further validate the FL release efficacy and the wireless implant functionality, we tested the system in adult male C57BL/6J mice. In these experiments, the DDM was inserted into a subcutaneous pocket in the upper back of an anesthetized mouse. The FL release was either powered by a potentiostat or the implant (Fig. 6a and b). For the implant case, the device was placed into a subcutaneous pocket in the lower back of the anesthetized mouse and powered *via* an externally located US transmitter.

The detected FL concentration in the mouse blood showed the voltage-triggered release of FL (Fig. 6c). At the start of the experiment (0 min), the DDM was inserted into the subcutaneous pocket. Ten minutes were then allocated to the alignment of the US transmitter to the implant (alignment period, “A”). From 10 min to 20 min, the implant was actuated and −1 V was applied to the DDM releasing the FL (stimulus period, “S”). The potentiostat experiments included the same alignment period for consistency but did not include US transmission. For the potentiostat control group, no voltage was applied to verify that the observed FL release was voltage triggered. The implant control experiment included the alignment and US transmission to the implant. The only difference was that the implant was disconnected from the DDM to verify that the US was not a confounding factor in the FL release. From 20 min onwards, the uptake of FL into the mouse's blood stream was monitored; no voltage was applied to the DDM during this time.

Consistent with the *in vitro* results (Fig. 5c), rapid on-demand FL release was observed upon electrical stimulus shown by the systematic FL levels in mice that reached approximately 80 ng mL^−1^ immediately after stimulus ended (Fig. 6c, blue solid line, 20 min). At the same time point, the control groups without potential applied showed traces of FL leaking with a mean blood level below 2 ng mL^−1^ (Fig. 6c, blue dashed line, 20 min). The circulating FL level gradually rose and peaked at 15 min post-stimulus with a mean value of 113 ng mL^−1^ (70–182 ng mL^−1^). Then, it slowly trended down and decreased to around 47 ng mL^−1^ (20–75 ng mL^−1^) at the end of the 2 h monitoring period.

When the implant was used to stimulate the DDM, FL release and absorption kinetics (Fig. 6c, red solid line) were almost identical compared to when the potentiostat was used as the stimulus source. Taken together with the results shown in Fig. 5c, the implant was successfully triggered while in the mouse and provided sufficient power and the expected electrical potential (−1 V), leading to a similar amount of FL release. The kinetics of the controls were also similar between the potentiostat and implant (Fig. 6c, dashed lines), showing that the ultrasound alone did not affect the leak rate.

Fluorescein has been extensively used as a tracer in routine ophthalmic tests in humans through intravenous injection. Fluorescein undergoes rapid metabolism once in circulation. Both FL and its metabolites are mainly eliminated *via* renal excretion. Subcutaneous injection of the FL solution at different dosages (Fig. S2†) showed a rapid absorption which peaked 5 min after injection followed by a fast clearance to less than 5% of the peak value at 120 min. In the potentiostat and implant study, we observed a delayed peak and slower clearance trend. The FL peak was 25–40 min after stimulus onset and was nearly 30% of the peak value at the end of the experiment. There were likely several reasons for the slower release and clearance. First, the FL release pattern from a 10 min stimulus was different from a bolus subcutaneous injection which lasts only seconds. Second, as shown in Fig. 4e, there was residual FL release from FL still in the process of migrating from the gel into the solution during the off period of the pulsed release. Third, the absorption of FL ions released from a DDM (without a counter ion) may be different than FL ions injected from a FL sodium solution.

In certain applications, acute release is preferred. For example, diabetic hypoglycemia onset requires fast release of anti-hypoglycemic drugs to restore blood glucose while also demanding its rapid decrease to avoid over-rescue resulting in hyperglycemia. In these cases, the implant can be placed in a region with abundant vascular supply, like the abdominal omentum, for accelerated absorption and a shortened stimulus time can be used to limit the drug release. In other situations when enhanced drug exposure effects are preferred to achieve biological effects, we can increase the drug releasing time frame and extend their exposure window by adopting a longer continuous powering period.

Area under the curve of FL concentration reflects the accumulated FL exposure of the mice. Both potentiostat and US triggered release were significantly higher than the control groups (Fig. 6d). This indicated that mice in the stimulus groups were exposed to much higher amounts of FL across the full observation span after electrode placement than the control groups regardless of whether the stimulation was from a potentiostat or the implant (*P* < 0.001).

In these experiments, the electrode and implant were separate components connected with wires. This was because the implant device could be reused across multiple experiments whereas each DDM was only used once for consistency across experiments. Separating the implant from the DDM allowed us to interchange electrodes without having to remake new devices for each experiment. In this implant design, the bottom did not contain any electronic components, and had space for the DDM electrodes. We do not foresee any fundamental challenges with combining the implant and DDM into one device.

Electronic control of DDMs can go beyond just triggering the electrical stimuli. While in this work we used a fixed voltage from the implant for consistent stimulation, there is far more potential to be tapped into in this platform. Additional circuits could be used to allow for programmable voltage stimuli. Using wireless downlink communication, the implant could receive commands to change the voltage levels in real time^21^ and modulate the release rate. By combining the system with dosage or other biomarker sensors, the release could be personalized in a closed-loop fashion.

## Conclusions

We developed an ultrasonically powered implant and DDM using an electronically controlled PPy nanoparticulate film. The battery-free mm-sized device is designed to receive power from an external ultrasonic transmitter allowing for minimally invasive implantation and wireless on-demand control of the DDM. The DDM is fabricated by coating an electrode with nanoparticles and a protective silicone oil-PDMS gel to maximize drug loading while minimizing passive release. The system was demonstrated using FL as a model drug. Release rate and kinetics for different stimulation voltages and number of pulses were measured *in vitro* to characterize the speed of release and the passive leak. Fluorescein release was also tested *in vivo* in mice, demonstrating wireless, controllable drug release. The measured release parameters were similar for stimuli generated using a benchtop potentiostat and the wirelessly powered implant. The results demonstrate the feasibility of ultrasound-powered implantable drug delivery using electroresponsive nanoparticles.

## Conflicts of interest

There are no conflicts of interest to declare.

## Supplementary Material

RA-012-D2RA03422K-s001
