# Supplementary material for: On-demand electrochemically controlled compound release from an ultrasonically powered implant
Source: RSC Adv. 2022 Aug 17;12(36):23337–45. doi: 10.1039/d2ra03422k (PMC9382542; doi:10.1039/d2ra03422k)
Supplement: RA-012-D2RA03422K-s001 [file RA-012-D2RA03422K-s001.pdf]

Electronic Supplementary Material (ESI) for RSC Advances.
This journal is © The Royal Society of Chemistry 2022

Electronic Supplementary Information

On-Demand Electrochemically Controlled Compound Release from an Ultrasonically Powered Implant

Max L. Wang[a,‡], Christian F. Chamberlayne[b,‡], Haixia Xu[c,‡], Mohammad Mofidfar[b,‡], Spyridon Baltsavias[a], Justin P. Annes[c], Richard N. Zare[b], Amin Arbabian[a]

[a] Department of Electrical Engineering, Stanford University, Stanford, CA, USA
[b] Department of Chemistry, Stanford University, Stanford, CA, USA
[c] Department of Medicine, Division of Endocrinology, Stanford University, Stanford, CA, USA
[‡] These authors contributed equally to this work.

Corresponding author email: arbabian@stanford.edu

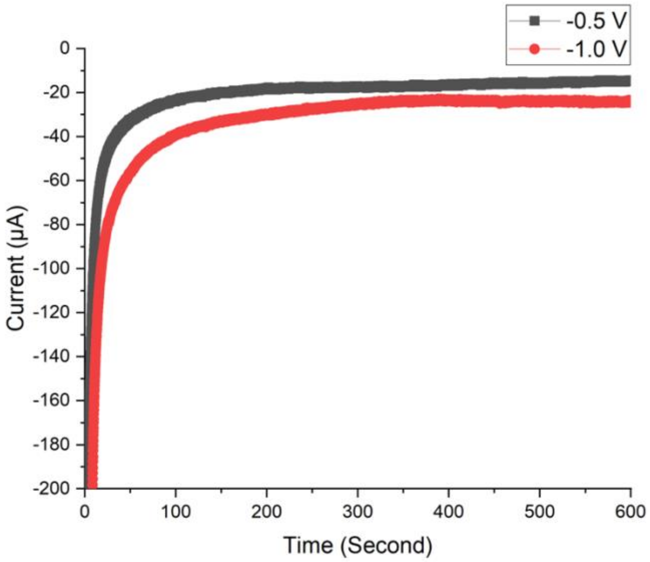

Fig. S1 Measured chronoamperogram of the fluorescein loaded nanoparticulate film during 10 min stimulation at -0.5 V (black) and -1 V (red).

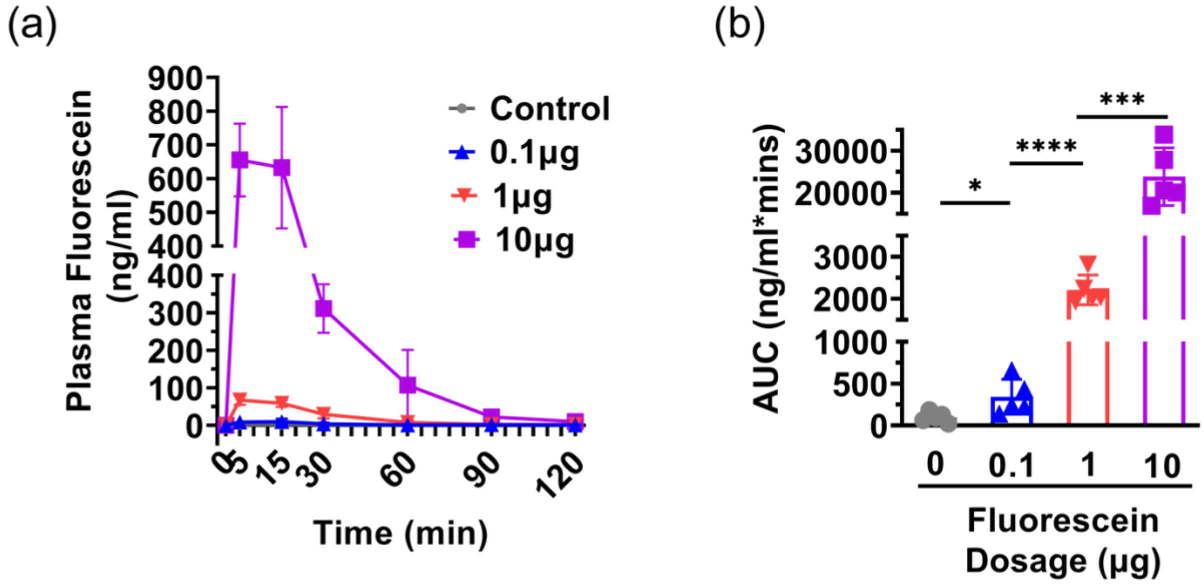

Fig. S2 (a) Dose curve of fluorescein after subcutaneous injection in male C57BL/6J mice (N=5/group) and (b) area under curve. Data represented as mean ± SD. *P<0.05, ***P<0.001, ****P<0.0001
